# Health-related quality of life and fatigue in HIV-1 infected patients diagnosed during primary versus chronic infection

**DOI:** 10.1186/1758-2652-13-S4-P1

**Published:** 2010-11-08

**Authors:** R Steingrover, MAF Nievaard, JMA Lange, PT Nieuwkerk, JM Prins

**Affiliations:** 1 Academic Medical Center, Amsterdam, Netherlands

## Background

Health-related quality of life (HRQOL) is affected by chronic HIV-infection (CHI). No data are available if and to what extent HRQOL is affected in patients who were diagnosed during primary HIV-1 infection (PHI).

## Methods

Included were Dutch, male, adult, HIV-1 infected patients, attending the AMC outpatient clinic who seroconverted after December 31st of 1996. 59 Patients identified during PHI and a randomly selected group of 99 patients that were identified with CHI in the same period were eligible. Patients were excluded if they ever had an CDC-C event or suffered any significant comorbidity. Subjects were asked to complete the SF-36 and the Multidimensional Fatigue Inventory (MFI-20) questionnaires. SF-36 and MFI-20 scores were compared with age- and gender-matched Dutch or German general population norms, respectively.

## Results

**Figure 1:**
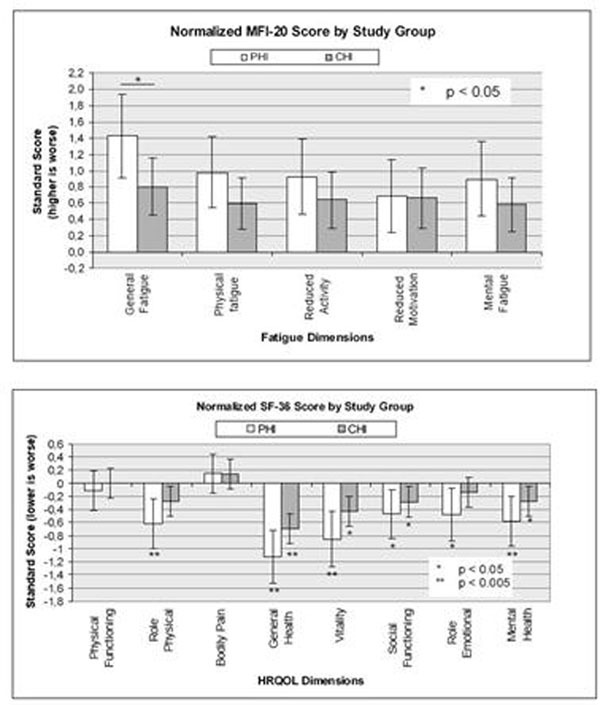


123 patients were included: 48 from the PHI group and 75 from the CHI group. Patients in the PHI group tended to be younger, fewer were on HAART and they had at the moment of diagnosis a higher CD4 count and a higher plasma viral load. The PHI group was more severely impaired and in more dimensions of HRQOL than the CHI group. Likewise, although fatigue was present in both PHI and CHI groups. PHI patients scored higher in every dimension. Figure 1

## Conclusions

HIV-infected patients in the HAART-era have severely impaired HRQOL and suffer from fatigue. Patients that presented with PHI in the past are impaired to a larger extent and in more dimensions than patients diagnosed with HIV during CHI. This difference is present in both HRQOL and fatigue.

